# Finite Element Analysis of Bone Stress around Micro-Implants of Different Diameters and Lengths with Application of a Single or Composite Torque Force

**DOI:** 10.1371/journal.pone.0144744

**Published:** 2015-12-14

**Authors:** Ying-juan Lu, Shao-hai Chang, Jian-tao Ye, Yu-shan Ye, Yan-song Yu

**Affiliations:** Department of Stomatology, Sun Yat-sen Memorial Hospital, Sun Yat-sen University, Guangzhou 510120, Guangdong Province, China; The Ohio State University, UNITED STATES

## Abstract

**Background:**

Stress on the bone surrounding dental micro-implants affects implant success.

**Purpose:**

To compare the stress on the bone surrounding a micro-implant after application of a single force (SF) of 200 g or a composite force (CF) of 200 g and 6 N.mm torque.

**Materials and Methods:**

Finite element models were developed for micro-implant diameters of 1.2, 1.6, and 2.0 mm, and lengths of 6, 8, 10, and 12 mm and either a SF or CF was applied. The maximum equivalent stress (Max EQS) of the bone surrounding the micro-implant was determined, and the relationships among type of force, diameter, and length were evaluated.

**Results:**

The Max EQS of the CF exceeded that of the SF (*P*< 0.05). The effect of force on stress was related to implant diameter, but not to implant length. The larger CF led to greater instability of the micro-implant and the effect was most pronounced at an implant diameter of 1.2 mm. The use of implant diameters of 1.6 mm and 2.0 mm produced no significant difference in implant stability when either a CF or SF was applied.

**Conclusion:**

When considering the use of an implant to perform three-dimensional control on the teeth, the implant diameter chosen should be > 1.2 mm.

## Introduction

Implants are being popular in orthodontic treatment, and efforts at improving their success rate have become an active area of research. Stress on bone around a micro-implant is an important factor influencing their success rate [1–4]. Factors such as the diameter and length of an implant and its direction of insertion in bone can affect the stress on the bone surrounding the implant and, subsequently, affect the success rate of the implant [5–8].

The upright tilt of molars has always been a difficult problem in orthodontic treatment. The center of resistance of the tooth is basically consistent with the geometric center of the root of the tooth [9–11]. The center of resistance of a multi-root tooth lies in the root furcation towards the direction of the root tip, and its location changes with the length of the root [9–11]. When a single force(SF)is imposed on the molar crown, a tipping movement occurs because the center of resistance is beneath the point of application. Thus, it is very difficult to achieve stability of molars that are tilted in both the mesiodistal and buccolingual directions with traditional methods. In recent years, it has been shown that imposing a composite force (CF) with torque to a micro-implant will result in motion of the tooth in three dimensions [9,10]. Guo et al. [11] used this method to change a tilted molar to a more upright position and found that it provided good dimensional control to a mesiobuccal tilted molar. They were also able to avoid the adverse effects of using other teeth as an anchorage[11]. However, no reports have examined the stress on the bone around a micro-implant when applying a composite force.

Finite element analysis (FEA) is commonly used in dental research to investigate the influence of various implant design factors (i.e., diameter and length of mini-screws, thread shape, thread size, degree of taper, effect of taper length on insertion torque, pullout strength, stiffness) on the stress distribution within the bone supporting the implants [12–17].

The purpose of this study was to use FEA to compare the stress on the bone around a micro-implant that occurs with two types of force, i.e., a CF containing torque vs.the traditional SF. The hypothesis underlying this study was that a larger CF would produce greater implant instability.

## Materials and Methods

### Establishment of the jaw model

This study was approved by the Institutional Review Board of Sun Yat-sen Memorial Hospital, Sun Yat-sen University. A signed IRB approved consent form was obtained from allhealthy volunteers before their data was used in the study.

Maxillary computed tomography (CT) data obtained from healthy volunteers, who were without dental or maxillofacial abnormalities, were saved in DICOM format and imported into Mimics 10.0 software (Macrovision Corporation, USA). Interference from neck and head structures was eliminated when performing the CT scan and interference from metal artifacts was removed at the same time. Using appropriate thresholds, soft and hard tissue structures were extracted and three-dimensional (3D) reconstructions of the hard and soft tissues were performed. The maxillary model was generated and Geomagic Studio 8.0 software (Raindrop Geomagic, USA) was used to refine the model. Finally, the model was exported in stp format for editing.

### Construction of the micro-implant model

The micro-implant model was based on the Tomas micro-implant (Dentaurum, Germany) as it is the most commonly used micro-implant. Unigraphics NX 6.0 software (Siemens Product Lifecycle Management Software, USA) was used to build the 3D model of the micro-implant. Models with diameters of 1.2 mm, 1.6 mm, and 2.0 mm and lengths of 6 mm, 8 mm, 10 mm, and 12 mm were created.

### Solid model assembly

The micro-implant was inserted into the buccal alveolar bone of the maxillary second molar at 10 mm from the mesial alveolar ridge, and 6 mm from the alveolar crest [11].The micro-implant placement orientation was 90°. The range in thickness of mandibular cortical bone was 1.5–2.0 mm.

### Generation of the 3D finite element model

The integrated plug in the ANSYS Workbench and Unigraphics was used to import the model to the ANSYS Workbench 13.0 (SAS, USA). Diameter and length parameters were simultaneously set by the analysis software. A tetrahedral mesh was used to divide the cortical bone, cancellous bone, and screws, and to improve the quality of the grid. The accuracy of the data was increased using the method of grid refinement for the junction of the micro-implant, cortical bone, and spongy bone. The model nodes ranged from 168,770 to 223,077 and the unit numbers ranged from 107,446 to 143,600.

### Material biomechanical parameters and mechanical loading

In this study, the type III bone classification of Lekholm and Zarb [18] was simulated. Material parameters are shown in Table 1. All materials were assumed to be homogeneous, isotropic, and linearly elastic. In previous studies, the bond between the micro-implant and jaw was set as fixed contact [2]. However, when considering an instantaneously applied load, there is only a mechanical bond between a micro-implant and the surrounding bone and mobility is present. For this reason, the bond between the implant and bone was considered to a frictional contact between the micro-implant and the jaw and the coefficient of friction was set at 0.3[5,12].The micro-implant modeled in this study was designed to bear a maximum single orthodontic force of 300 g and an instantaneous load value not exceed 200 g [19,20].

**Table 1:** Material properties of the bone and micro-implant.

Material	Young’s modulus (MPa)	Poisson’s ratio
Cortical bone	13.70	0.33
Cancellous bone	1,600	0.3
Mini-implant	110,000	0.35

**Fig 1:**
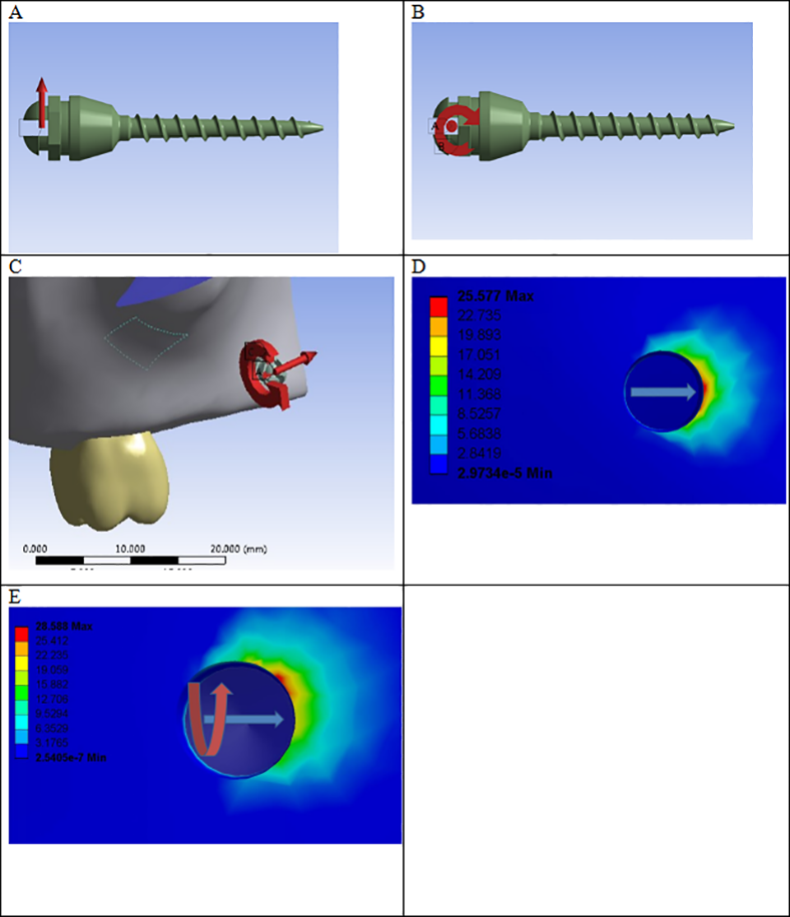
Illustrations of forces applied to the implant in the model. (A) Single force. (B) Torque. (C) Composite force. (D) Single force stress disturbances. The circle is the cross section of micro-implant. The micro-implant with a diameter of 1.2 mm and a length of 6 mm has equivalent stress disturbances on the surrounding cortical bone. The arrow refers to horizontal force, i.e., the single force (SF). (E) Composite force stress disturbances. The circle is the cross section of a micro-implant. The micro-implant with a diameter of 1.2 mm and a length of 6 mm has equivalent stress disturbances on the surrounding cortical bone. The composite force is the superposition of the single force and torque force. The horizontal arrow refers to the single force and the curved arrow refers to the torque force.

Two simulated forces were used: (1) a SF of 200 g loaded near the middle, and(2) a CF of 200 g and 6 N^**.**^mm torque in the cross grove (Fig 1). As each sized micro-implant corresponded to eight models, fthemodels were chosen randomly for unidirectional force loading, while the other fthemodels were chosen for composite force loading.

### Evaluation indices

The maximum equivalent stress (Max EQS) of cortical bone and cancellous bone were determined by varying the diameter and length of the implant and force applied to the implant (single and composite). A lower Max EQS suggested that the stress on the bone surrounding the implant would be lower, the possibility of being damaged would be less and the success rate would be higher[19–21].

### Statistical analysis

Continuous variables were presented as mean ± standard deviation (SD), and were compared using the means. As the correlation between the Max EQS of cortical bone and cancellous bone was significant (Pearson’s r = 0.30, *P* = 0.003), a multivariate factorial analysis of variance (MANOVA) was utilized to examine between-group effects. A two-tailed *P*-value< 0.05 was considered statistically significant. All analyses were performed using SPSS Version 20 (SPSS Statistics V20, IBM Corporation, Somers, New York).

## Results

**Fig 2:**
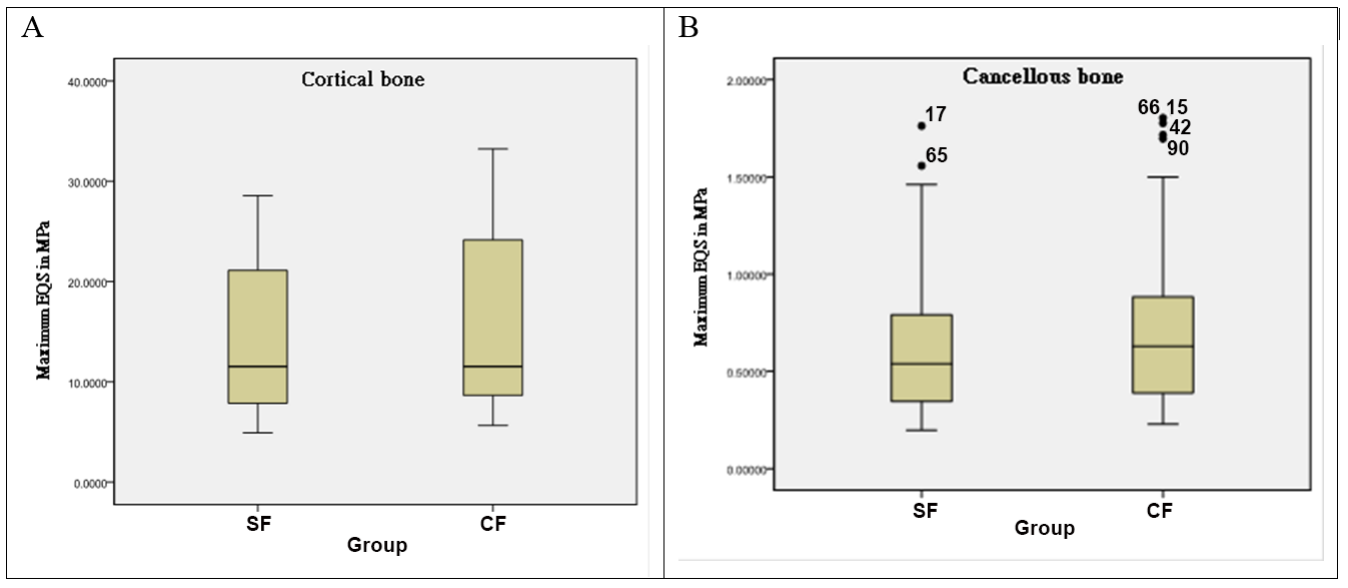
Maximum equivalent stress (Max EQS) in (A) cortical bone and (B) cancellous bone with application of a single force (SF) and composite force (CF).

The Max EQS,on both the cortical and cancellous bone surrounding the implant, after application of a SF and composite force is shown in Fig 2. The results showed that when a CF was applied,Max EQS was slightly higher than when a SF was applied and the effect was more pronounced in cancellous bone.

Results of the MANOVA analysis of between-group effects are shown in Table 2. No three-way interaction was found in either cortical or cancellous bone. One two-way interaction of cancellous bone was present (diameter × length), but there was no interaction with force direction. Two two-way interactions of cortical bone were also present (force × diameter and diameter × length). These results indicated that the direction of the force significantly affected the Max EQS in cancellous bone (*P*< 0.001).

**Table 2:** MANOVA analysis of between-group effects.

Sthece	Cortical bone		Cancellous bone	
	*F*	*P*	*F*	*P*
Force	9.81	0.003	14.10	<0.001
Diameter	621.48	<0.001	50.24	<0.001
Length	11.30	<0.001	203.96	<0.001
Force × diameter	6.09	0.004	0.45	0.642
Force × length	0.83	0.484	0.86	0.465
Diameter × length	2.57	0.026	46.37	<0.001
Force × diameter × length	0.55	0.790	0.25	0.960

**Fig 3:**
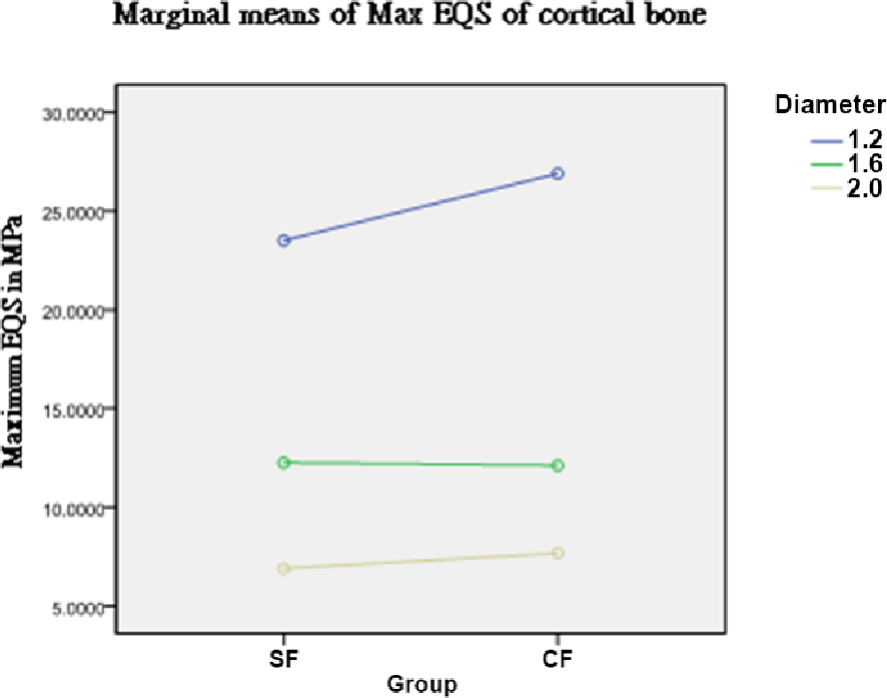
Interaction of force type and implant diameter on Max EQS in cortical bone. SF, single force; CF, composite force.

Since the interaction of force and diameter was significant in cortical bone (*P* = 0.004), the effect of the type of force at different diameters was examined. As seen in Table 3 and Fig 3, the Max EQS differed significantly with the two types of forces using an implant with a diameter of 1.2 mm (*P* = 0.007).

**Table 3:** Independent effect of force on maximum equivalent stress (Max EQS)in cortical bone by implant diameter.

Implant Diameter (mm)	Force (MPa)	Mean ± SD	*t*	*P*
1.2	Single	23.50 ± 2.76	-2.89	0.007
	Composite	26.89 ± 3.79	-2.89	0.007
1.6	Single	12.26 ± 2.60	0.149	0.882
	Composite	12.13 ± 2.31	0.149	0.882
2.0	Single	6.94 ± 1.11	-1.748	0.091
	Composite	7.70 ± 1.35	-1.748	0.091

## Discussion

This study compared the stress on the bone surrounding a micro-implant of varying diameters after application of a SF of 200 g or a CF of 200 g and 6 N.mm torque. The results showed that a larger CF led to greater instability of the micro-implant and the effect was most pronounced at an implant diameter of 1.2 mm.

A lower Max EQS indicated less stress on the surrounding bone, which implied less damage to the bone and, subsequently, a higher micro-implant success rate [3,6,22]. The results of this analysis also suggested an interactive effect between the implant diameter and the applied force since the CF exhibited different effects depending on implant diameter. When the diameter of the implant was 1.2 mm, a CF resulted in greater implant instability, while the use of implant diameters of 1.6 mm and 2.0 mm produced no significant change in implant stability when either a CF or SF was applied. This suggests that the use of a larger diameter implant is preferred when stability is desired. However, too large a diameter would be associated with increased surgical trauma to the bone. Further studies are necessary to determine the optimal cut-off diameter that maximizes stability while minimizing trauma due to insertion.

Other studies have evaluated the relationship between the micro-implant diameter and its initial stability. The studies of Holm et al. showed that the Max insert torque (MIT) of a micro-implant with diameter of 2.0 mm was greater than the MIT measured with implant diameter of 1.5 mm, i.e., the initial stability was stronger with the larger implant [23], similar to the findings. Similarly, Smith et al. tested the torque of a micro-implant of varying diameters ranging from 1.4 to 1.8 mm and also concluded that the larger the diameter, the stronger of the initial stability. Lin et al. proved, by 3-D FEA, that the larger diameter of the implant, the less cortical bone stress, and the stronger of the initial stability [24]. Under the effect of two loading conditions, the study showed that the implant cortical bone stress peak decreased as the diameter was increased, i.e., the larger the diameter, the better the initial stability.

Previous studies have also examined the association between characteristics of the implant and stress on the surrounding bone. Similar to the findings, Duaibis et al. [6] found (when using FEA to evaluate factors affecting the stress in cortical bone surrounding mini-screw implants)that implant diameter affected the stress within the bone around the implant, while thread shape, thread pitch, and cortical bone thickness did not. However, contrary to the findings, they also showed that implant head length, (along with thread size and elastic modulus of cancellous bone) also affected stress within the bone surrounding the implant. Kang et al. [8] used FEA to simulate the stress distribution of 8-mm implants with six different diameters in type I to IV bone. Their results showed that the maximum Von-Mises stress varied significantly when the diameter was between 3.3 mm and 5 mm. However, they found when the diameter was increased from 5.5 mm to 7.1 mm, the peak stress on the implant-bone interface increased with the reduction in bone density. Coelho Goiato et al. [7] examined the stress associated with implants of 2.5 mm, 3.3 mm, and 3.75 mm in diameter and found that the 3.75 m implant was associated with a better stress distribution than the other two implants studied.

Regarding the effect of torque on the posterior teeth, Chan et al. [21] and Hohmann et al. [25] showed that a torque > 6 N^**.**^mm will cause root resorption. Thus, a single force of 200 g and a composite force of 6 N.mm used in this study simulated the loads typically imposed during the clinical insertion of a micro-implant. Furthermore, although the majority of biological materials are non-uniform and anisotropic, this study showed that when only small deformations are examined, finite element models can be used assuming the materials are continuous, homogeneous, isotropic, and linearly elastic [26].

The primary limitation of this study was that its results were derived using a model and, thus, may not be applicable to the clinical setting. In addition, further studies are necessary to determine an optimal cut-off diameter that maximizes stability and minimizes insertion trauma. In previous studies involving dental orthopaedics, the micro-implant functioned only as reinforcement of anchorage and controlled the movement of teeth in only one direction, while in this study, rectangular wires were used to directly connect the micro-implant to the teeth, simplifying the composition of system used for correction. Since the effect of rectangular wires on the micro-implant also includes torque force imposed by the groove at the top (in addition to the conventional horizontal force) the stability of micro-implant becomes unknown. Future studies, therefore, are needed to assess this issue.

The previous literature primarily analyzed the stability of the micro-implant under a single force. The study analyzed the initial stability of the micro-implant under a composite force containing torque. The results showed that under the composite force of torque and horizontal force, the varied-diameter implant displacement peaks were larger than those due to horizontal force alone. The results showed that the composite interaction of torque and horizontal force on the implant can have adverse effects on the initial stability of the implant, and herein lies the contribution of the study to the orthodontic literature.

In conclusion, this study showed, using FEA, that a larger CF led to greater instability of the micro-implant and the effect was most pronounced for an implant diameter of 1.2 mm. However, using implant diameters of 1.6 mm and 2.0 mm produced no significant difference in implant stability when either a CF or SF was applied. Therefore, when considering the use of an implant to perform 3-D control on the teeth, the implant diameter should be > 1.2 mm. However, too large a diameter will be associated with increased surgical trauma to the bone. Further studies are, therefore, necessary to determine the optimal cut-off diameter that maximizes stability while minimizing insertional trauma.
